# Age-Friendly Palliative Care for Veterans and Caregivers in a Rural VA Healthcare System -- the Eastern CO Experience

**DOI:** 10.1093/geroni/igaf122.3154

**Published:** 2025-12-31

**Authors:** Stephanie Hartz, Alexander Garbin, Courtney McGuire, Jill Steagall, Jocelyn McCauliff, Kathryn Nearing

**Affiliations:** VA Eastern Colorado Health Care System, Aurora, Colorado, United States; Eastern Colorado VA Health Care System, Aurora, Colorado, United States; Geriatric Research Education and Clinical Center, Aurora, Colorado, United States; Rocky Mountain Regional VA Medical Center, Aurora, Colorado, United States; Veterans Affairs, Aurora, Colorado, United States; University of Colorado Anschutz Medical Campus, Denver, Colorado, United States

## Abstract

**Introduction:**

Age Friendly Health Systems (AFHS) provide care to older adults aligned with geriatric best practices to improve healthcare quality, minimize harms and support older adults’ goals of care. AFHS designation means that providers consistently address the Geriatric 4Ms during clinical visits: Mentation, Mobility, Medication and What Matters Most.

**Methods:**

Within the VA Eastern Colorado Health Care System (ECHCS), our rural tele-Palliative Care clinic achieved AFHS Level 2 Committed to Care Excellence recognition in December 2021, becoming the first AFHS-designated telemedicine clinic in the nation. We engaged older Veterans in planning and implementation. Using our existing interprofessional team and clinic workflow, we addressed the Geriatric 4Ms during visits.

**Results:**

FY23-24, we conducted 192 AFHS tele-Palliative Care visits, 81% with rural/highly rural Veterans. We served 108 unique patients (FY23: 57; FY24: 51). Compared to Colorado’s Veteran population, racially minoritized Veterans and women were underrepresented, while older Veterans were overrepresented. The majority were White (82% and 73%), not Hispanic/Latino (83% and 73%), male (100% and 98%), and ≥65 (90% and 89%). All 4Ms were addressed for 86% (FY23) and 76% (FY24) of unique patients. AFHS tele-Palliative Care saved Veterans/caregivers 23,622 (FY23) and 18,632 (FY24) miles of travel.

**Conclusion:**

Congruent with AFHS, Palliative Care focuses on physical, emotional, and psychosocial aspects of serious illness. Our work to achieve AFHS Level 2 designation in a tele-Palliative Care clinic is novel nationally. We demonstrated that evidence-based care can be provided to every older adult, regardless of care modality, without expanding staff or changing clinical workflows.

